# Molecular Landscape of *ERBB2* Alterations in 14,956 Solid Tumors

**DOI:** 10.3389/pore.2022.1610360

**Published:** 2022-07-13

**Authors:** Hao Wang, Ji Miao, Yazhou Wen, Xihua Xia, Yanan Chen, Mengli Huang, Shiqing Chen, Zhengyi Zhao, Yuzi Zhang, Chunzhu Chen, Xinhua Zhu

**Affiliations:** ^1^ Department of General Surgery, Nanjing Drum Tower Hospital, The Affiliated Hospital of Nanjing University Medical School, Nanjing, China; ^2^ Department of Anesthesiology, Women’s Hospital of Nanjing Medical University, Nanjing Maternity and Child Health Care Hospital, Nanjing, China; ^3^ The Medical Department, 3D Medicines Inc., Shanghai, China

**Keywords:** next-generation sequencing, TMB, *ERBB2*, solid tumors, anti-HER2 agents

## Abstract

*ERBB2* abnormalities frequently occur and serve as rationale therapeutic targets in cancer. In this study, clinical and next-generation sequencing data from 14,956 patients across more than 20 tumor types were collected. A total of 406 (2.7%) patients were identified with *ERBB2* amplifications, and 303 (2.0%) patients with pathogenic somatic *ERBB2* mutations. *ERBB2* amplifications fell most frequently in breast (15.9%) and stomach (8.3%) cancers. Somatic *ERBB2* SNVs/indels occurred most common in bladder/urinary tract (7.3%) and intestine (6.1%) cancers. The top mutated *ERBB2* SNVs/indels were p.Y772_A775dup (25.5%) and p.S310F/Y (19.9%). Significantly higher rates of *ERBB2* SNV/indels were found in women compared to men (2.8% vs. 1.5%, *p* < 0.0001). *CDK12* was the most common co-amplification gene with *ERBB2* in cancers with a high frequency of *ERBB2* amplifications. Patients with *ERBB2* amplifications or mutations had higher TMB compared with patients with non-*ERBB2* alterations. The study provided the landscape of *ERBB2* alterations across a variety of solid tumors that may benefit from anti-HER2 agents.

## Introduction

*ERBB2* gene, also known as *HER2*, is a well-known proto-oncogene encoding a receptor tyrosine kinase (RTK), which consists of an impaired extracellular ligand-binding structure containing two receptor-L domains and a furin-like cysteine-rich region, a single hydrophobic transmembrane domain and an intracellular tyrosine kinase domain (1–3). Alterations of *ERBB2* including gene amplification, protein overexpression, and missense mutations have been reported in multiple solid cancers, especially in breast cancer (4), stomach cancer (5), and non-small cell lung cancer (NSCLC) (6). These aberrant activations of *ERBB2*, independent of ligand-receptor stimulation, promote tumorigenesis, tumor growth, and progression (7).

Since the first monoclonal antibody targeting the extracellular domain of HER2, Trastuzumab, got approved by the US Food and Drug Administration (FDA) for the treatment in HER2-positive breast cancer in 1998, a number of anti-HER2 agents have been developed and modified for the treatment of patients harboring *ERBB2* amplifications, including monoclonal antibodies, small molecular drugs, and antibody-drug conjugates (ADCs). Trastuzumab in combination with chemotherapy has been approved for the first-line treatment of patients with HER2-positive metastatic gastric cancer since 2009 (8). The tyrosine kinase inhibitor lapatinib can bind to the ATP-binding pocket reversibly and suppress the *RAS/RAF/MEK/ERK* and PI3K-AKT signaling in HER2-positive solid cancers (9, 10). In recent years, the bispecific antibodies that covalently combine cytotoxic agents and monoclonal antibodies, i.e., antibody-drug conjugates (ADCs), have emerged as a novel class of targeted anti-cancer drug. Several ADCs derived from anti-HER2 mAbs have exhibited excellent clinical efficacy and have been approved for patients with solid cancers with *ERBB2* amplification (11–15). On the other side, although no targeted drugs are approved for *ERBB2*-mutated cancers by far, several clinical trials have exhibited promising results in lung cancer patients carrying *ERBB2* mutations. In a single-arm, open-label, phase II study, Poziotinib, an irreversible pan-HER inhibitor, showed hopeful antitumor activity with an ORR of 27% in patients with *ERBB2* exon 20 mutant NSCLC including patients who had previously received platinum-based chemotherapy. The median progression-free survival (mPFS) was 5.5 months (95% CI, 4.0–7.0) and the median overall survival (mOS) was 15 months (95% CI, 9.0 to not estimable)(16). Based on the encouraging clinical efficacy of Poziotinib, FDA granted it fast-track designation. Results from a phase II basket trial displayed that Ado-trastuzumab emtansine provided a confirmed partial response rate of 44% (95% CI, 22%–69%) in 18 patients with advanced HER2-mutant lung adenocarcinomas and mPFS was 5 months (95% CI, 3–9 months). Responses were seen in patients with *ERBB2* exon 20 insertions and point mutations in the kinase, transmembrane, and extracellular domains (17). Most recently, a multicenter, international, phase 2 study revealed that Trastuzumab deruxtecan showed durable antitumor activity with ORR of 55% in 91 patients with previously treated HER2-mutant NSCLC. mPFS was 8.2 months (95% CI, 6.0–11.9), and mOS was 17.8 months (95% CI, 13.8–22.1) (18). Therefore, the National Comprehensive Cancer Network (NCCN) guidelines for non-small cell lung cancer (NSCLC) recommend that testing should be conducted as part of broad molecular profiling to detect *ERBB2* mutations in newly diagnosed patients and Ado-trastuzumab emtansine and Trastuzumab deruxtecan are available targeted agents in *ERBB2*-mutated NSCLC.

In the present study, we aim to explore the molecular landscape of *ERBB2* alterations in solid tumors by next-generation sequencing (NGS), which will provide additional evidence for the panorama of these therapeutic targets in the Chinese cancer population.

## Materials and Methods

### Samples

Formalin-fixed paraffin-embedded (FFPE) samples from cancer patients who underwent NGS between January 2017 and June 2020 were screened for analysis. The clinicopathological information from the patients and their sequencing data were collected.

This study was conducted under the approval of the ethics committees of the hospitals and informed consent was obtained from patients.

### NGS Detection

The methods for genome profiling have been described in the previous publication (19). Briefly, genomic profiling of DNA was performed through NGS with a >150-gene panel on Illumina Nextseq 500 to >500X coverage in 3DMed Clinical Laboratory Inc., a laboratory certified by both College of American Pathologists (CAP) and Clinical Laboratory Improvement Amendments (CLIA). Both somatic and germline alterations were analyzed, including single nucleotide variants (SNVs), insertion and deletions (indels), and copy number variants (CNVs). All SNVs and indels in the coding region of targeted genes, including missense, silent, stop gain, stop loss, in-frame, and frameshift mutations were considered. The CNVs of tumor tissues were calculated by BIC-seq2 (20). In addition, the TMB was defined as the number of nonsynonymous somatic SNVs and indels in examined coding regions, with driver mutations excluded. A microsatellite instability (MSI) score was defined as the percentage of unstable loci. Any sample with an MSI score of ≥0.4 was classified as MSI-H, and otherwise MSS. The identification of pathogenic levels of variants was obtained based on the published reports and the recommendation of the American College of Medical Genetics and Genomics and the Association for Molecular Pathology (ACMP-AMP).

### Statistical Analysis

Categorical variables were described as number and proportions. Categorical relationships were examined by using Pearson’s chi-square test with the Yates continuity correction when applicable and *p* value <0.05 was considered statistically significant. The SPSS22.0 software (SPSS, Inc., Chicago, IL, United States) was carried out for statistical analysis.

## Results

A total of 14,956 cancer patients who have received tissue NGS with a panel of more than 150 cancer-related genes were included for analysis. The median age was 59 (IQR range, 10–94), and 60% were men. The patients carried more than 20 types of solid tumor, including lung cancer (*n* = 4,624, 30.9%), liver cancer (*n* = 2,021, 13.5%), colorectal cancer (*n* = 1,581, 10.6%), stomach cancer (*n* = 998, 6.7%), biliary tract cancer (*n* = 840, 5.6%), etc. Among 12,450 patients who were evaluable for MSI status, 258 (1.7%) presented with MSI-H (Table 1).

**TABLE 1:** Clinicopathological information of the cancer patients.

Characteristics	All patients (*N* = 14,956)
Age, median (IQR range)	59 (10–94)
Sex, n (%)	
Male	8,980 (60.0)
Female	5,976 (40.0)
MSI status, n (%)	
MSI-H	258 (1.7)
MSS	12,192 (81.5)
N/A	2,506 (16.8)
Tumor types, n (%)	
Lung cancer	4,624 (30.9)
Liver cancer	2021 (13.5)
Colorectal cancer	1,581 (10.6)
Stomach cancer	998 (6.7)
Biliary tract cancer	840 (5.6)
Kidney cancer	828 (5.5)
Pancreas cancer	778 (5.2)
Breast cancer	503 (3.4)
Sarcoma	437 (2.9)
Ovary cancer	394 (2.6)
Head and Neck Cancer	340 (2.3)
Others	306 (2)
Cervical cancer	225 (1.5)
Esophagus cancer	195 (1.3)
Intestine cancer	179 (1.2)
Bladder/urinary tract cancer	177 (1.2)
Endometrium cancer	152 (1)
Prostate cancer	131 (0.9)
Neuroendocrine carcinoma	128 (0.9)
Gastrointestinal stromal tumors	119 (0.8)

**FIGURE 1:**
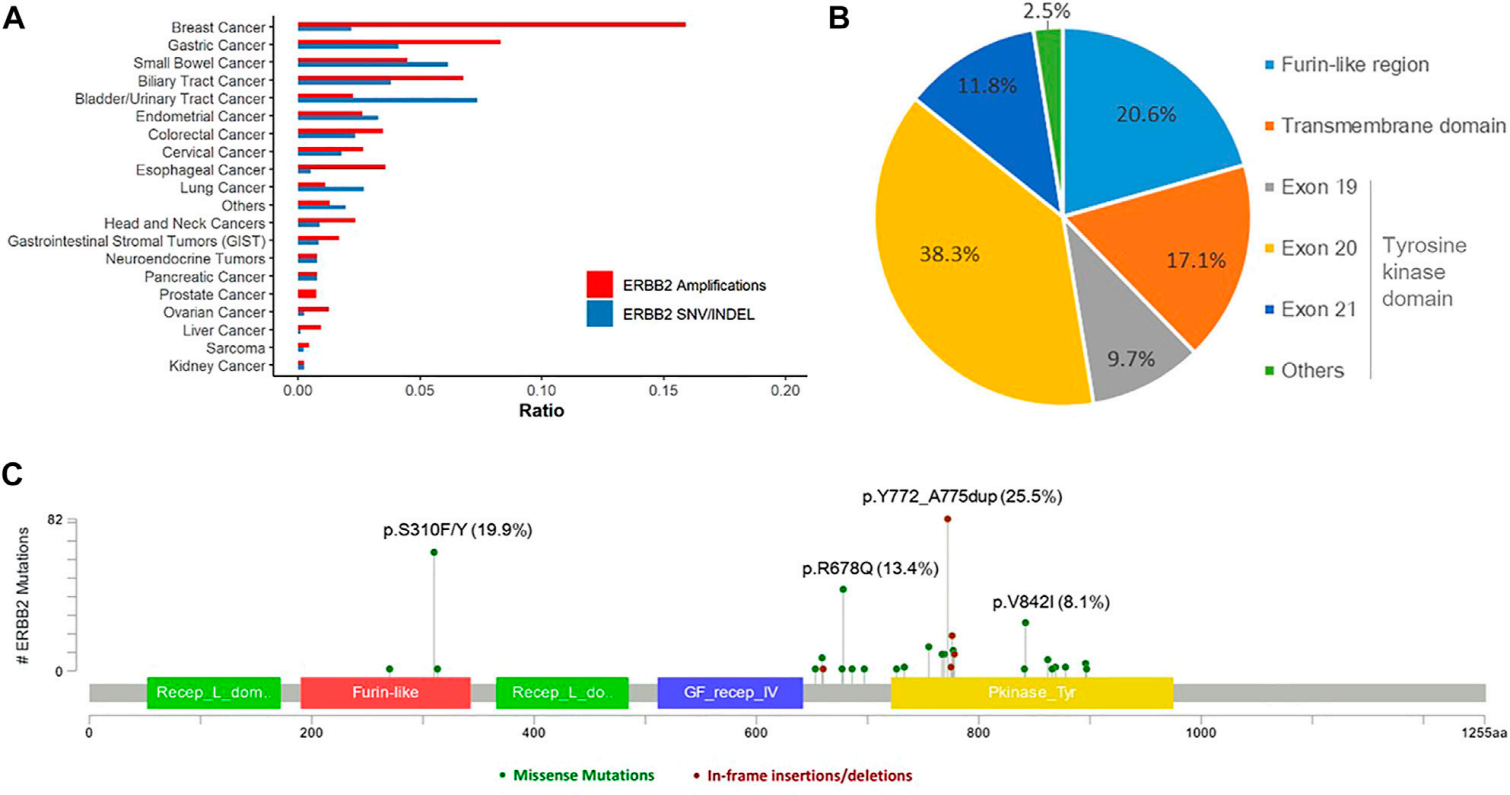
Landscape of *ERBB2* alterations. **(A)** Landscape of *ERBB2* amplifications and *ERBB2* mutations across different tumor types. **(B)** Prevalence of the pathogenic or very likely pathogenic somatic mutations of *ERBB2* in furin-like region, transmembrane domain, and tyrosine kinase domain. **(C)** *ERBB2* somatic mutation map.

Overall, 303 (2.1%) patients were identified with 321 pathogenic or very likely pathogenic somatic *ERBB2* SNVs/indels, including 213 missense mutations and 108 in-frame insertions or deletions, identifying 46 unique SNV/indel mutations. In addition, germline *ERBB2* alterations were found in 157 (1.0%) patients. Amongst all, 40 patients harbored at least two somatic *ERBB2* mutations, including 25 with concomitant *ERBB2* amplifications and mutations. Somatic *ERBB2* SNVs/indels occurred most common in bladder/urinary tract cancer (13/177, 7.3%), intestine cancer (11/179, 6.1%), stomach cancer (41/998, 4.1%), endometrium cancer (5/152, 3.3%), and lung cancer (125/4,624, 2.7%). Of note, SNVs were the majority of variants in almost all the cancers, except for lung cancer in which indels were the main variants. On the other side, the mutational frequency of *ERBB2* was the lowest in kidney cancer, sarcoma, liver cancer, and ovary cancer (Figure 1A). Across all cancers, *ERBB2* mutations occurred most frequently in the tyrosine kinase domain (62.3%) which included mutations in exon 19 (9.7%), exon 20 (38.3%), exon 21 (11.8%), and others (2.5%), followed by a furin-like cysteine-rich region of the extracellular domain (20.6%) and transmembrane domain (17.1%, Figure 1B). Of note, *ERBB2* exon 20 insertions were detected in 93 (0.6%) patients, accounting for 29.0% of all detected *ERBB2* SNV/indels, including 86 with lung cancer, two with pancreas cancer, one with breast cancer, etc. The top mutated *ERBB2* SNVs/indels were *ERBB2 p.Y772_A775dup* (82/321, 25.5%), a type of in-frame insertion within the exon 20 of the tyrosine kinase domain, and *p.S310F/Y* (64/321, 19.9%) in furin-like cysteine-rich region, followed by *p.R678Q* (43/321, 13.4%) in the transmembrane domain, and *p.V842I* (26/321, 8.1%) in the tyrosine kinase domain, etc. (Figure 1C; Table 2). In addition, the form and frequency of each *HER2* variant was observed for different tumor sites (Supplementary Table S1).

**TABLE 2:** Prevalence of the pathogenic or very likely pathogenic somatic mutations of *ERBB2*.

SNV/indels	Counts	Percent (%)
*p.Y772_A775dup*	82	25.5
*p.S310F*	47	14.6
*p.R678Q*	43	13.4
*Others*	27	8.4
*p.V842I*	26	8.1
*p.S310Y*	17	5.3
*p.V777L*	11	3.4
*p.G776delinsVC*	10	3.1
*p.L755S*	9	2.8
*p.G778_P780dup*	8	2.5
*p.I767M*	8	2.5
*p.D769Y*	7	2.2
*p.V659E*	7	2.2
*p.T862A*	5	1.6
*p.G776V*	3	0.9
*p.L755P*	3	0.9
*p.D769H*	2	0.6
*p.H878Y*	2	0.6

**FIGURE 2:**
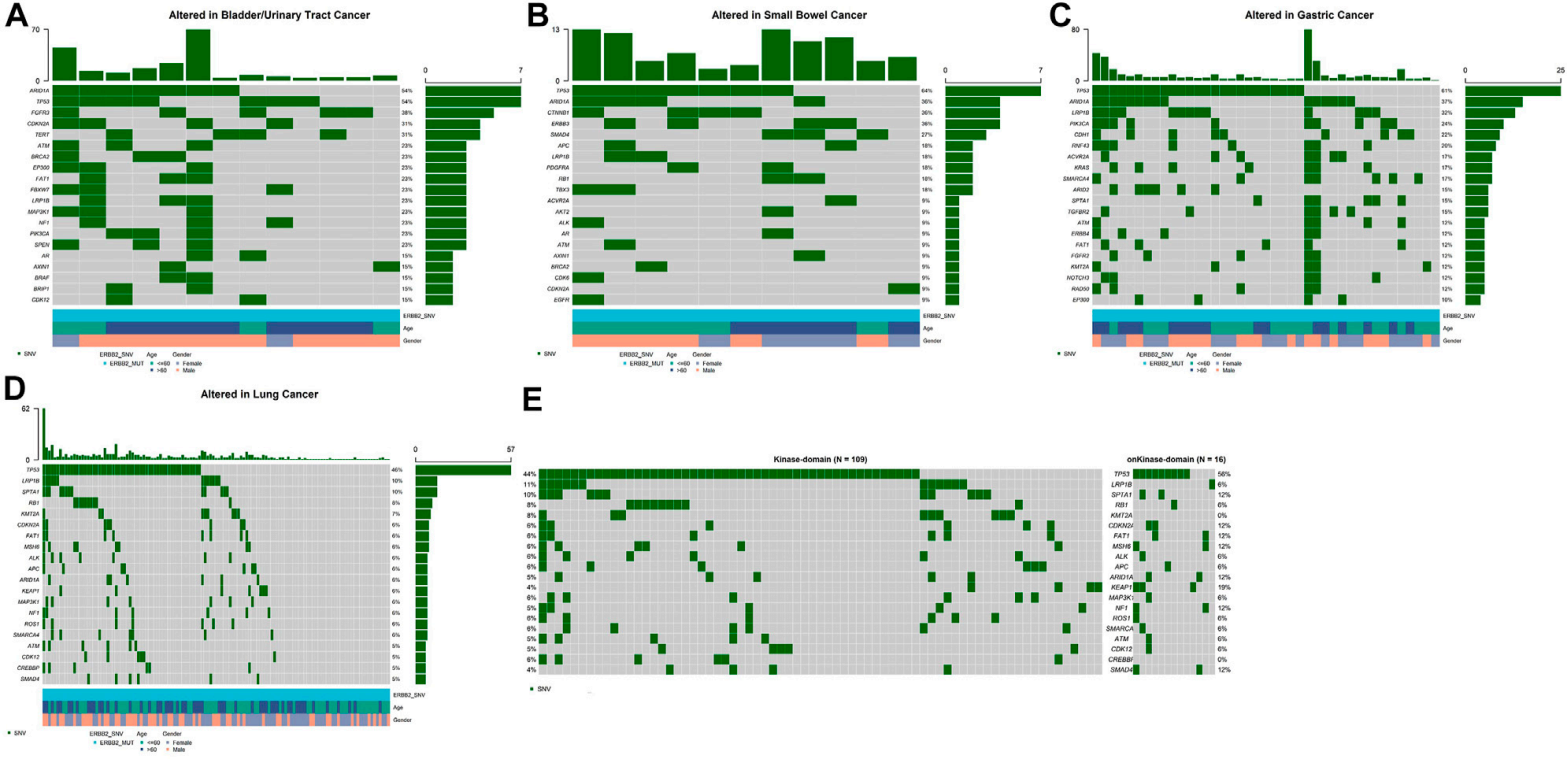
Top 20 high-frequency mutated genes in *ERBB2*-mutated patients of bladder/urinary tract cancer **(A)**, intestine cancer **(B)**, stomach cancer **(C)**, lung cancer **(D)**, kinase domain, and non-kinase domain in lung cancer **(E)**.

Among several cancers in which *ERBB2* mutation occurred most common, we investigated the genetic variation spectrum. In bladder/urinary tract cancer, *ARID1A* (54%), *TP53* (54%), and *FGFR3* (38%) were the high-frequency mutated genes (Figure 2A), whereas *TP53* (64%), *ARID1A* (36%), and *CTNNB1* (36%) were common genes observed in intestine cancer (Figure 2B). Furthermore, *TP53* (61%), *ARID1A* (37%), and *LRP1B* (32%) were the top three mutated genes in stomach cancer (Figure 2C), while in lung cancer, *TP53* (46%), *LRP1B* (10%), and *SPTA1* (10%) were the most frequently mutated genes (Figure 2D). We further explored whether *ERBB2* mutation in different domains would lead to a difference in mutation frequency in the variation spectrum. Analysis indicated that *CDKN2A*, *FAT1*, *MSH6*, *ARID1A*, *KEAP1*, *NF1*, and *SMAD4* mutation was more recurrent in patients with *ERBB2* SNV/indels within the kinase domain compared with non-kinase domain in lung cancer (Figure 2E).

**FIGURE 3:**
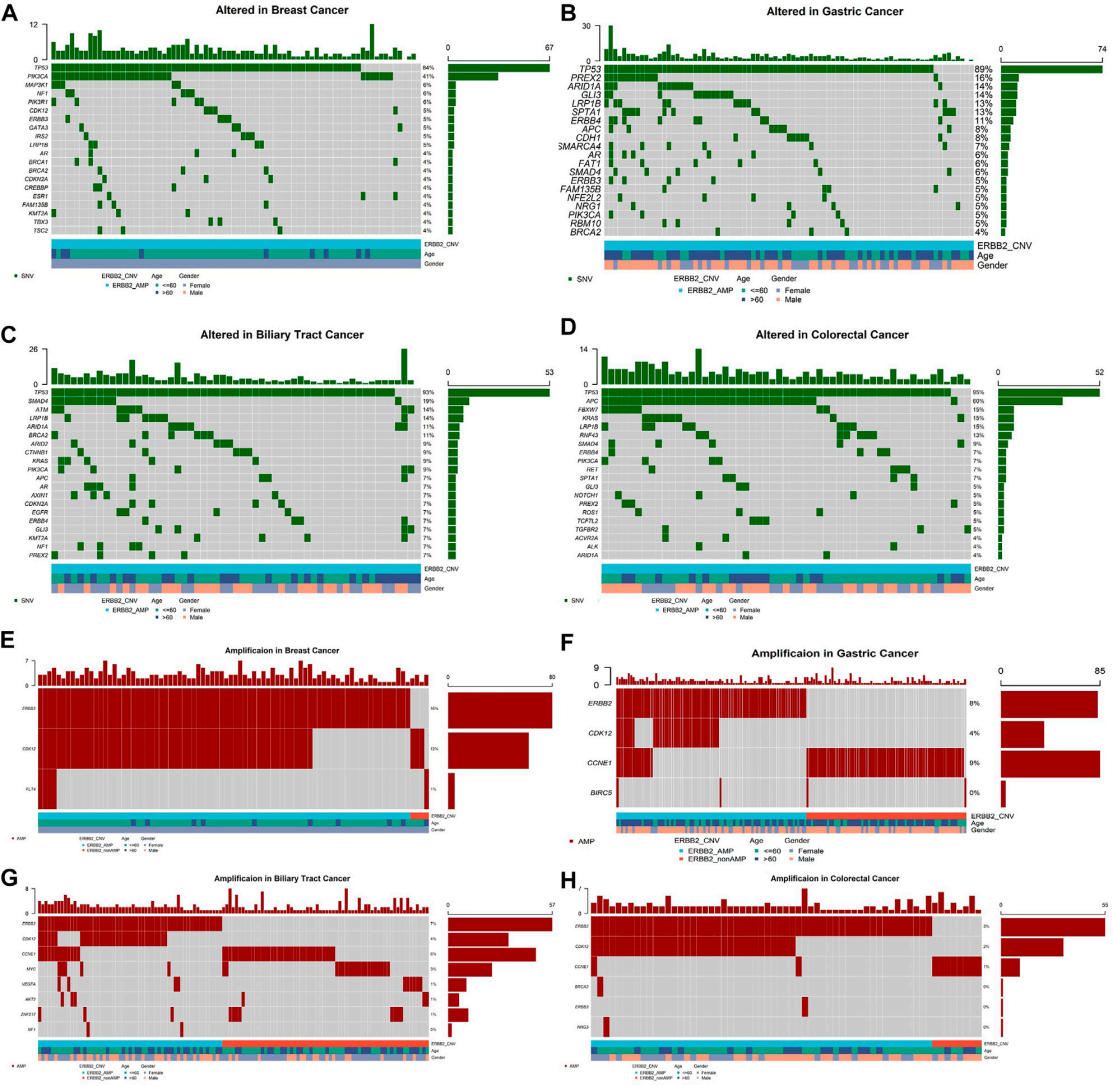
Top 20 high-frequency mutated genes in *ERBB2*-amplification patients of breast cancer **(A)**, stomach cancer **(B)**, biliary tract cancer **(C)**, colorectal cancer **(D)**, and co-amplification genes accompanying *ERBB2* amplification in breast cancer **(E)**, stomach cancer **(F)**, biliary tract cancer **(G)**, colorectal cancer **(H)**.

Meanwhile 406 (2.7%) patients were identified with *ERBB2* amplifications. *ERBB2* amplifications fell most frequently in breast cancer (80/503, 15.9%), stomach cancer (83/998, 8.3%), biliary tract cancer (57/840, 6.8%), intestine cancer (8/179, 4.5%), and esophagus cancer (7/195, 3.6%, Figure 1A). Among *ERBB2*-amplification patients, except for TP53 which was the top most mutated gene, *PIK3CA* ranked second of high frequency mutated genes in breast cancer (Figure 3A) and mutation of *PREX2* was recurrent in stomach cancer (Figure 3B). In biliary tract cancer, *SMAD4* was the more frequent mutated gene (Figure 3C). *KRAS* showed a decreased mutation frequency in colorectal cancer patients with *ERBB2* amplification (Figure 3D) compared with non-*ERBB2* amplification (15% vs. 49%). On the other hand, *CDK12* was the most common co-amplification gene with *ERBB2* in breast cancer (59/80, 73.8%, Figure 3E), stomach cancer (37/83, 44.6%, Figure 3F), biliary tract cancer (33/57, 57.9%) (Figure 3G) and colorectal cancer (33/55, 60%, Figure 3H).

No association was observed between the incidence of *ERBB2* mutations and age (>60 vs. ≤60, *p* = 0.95 for SNVs/indels and *p* = 0.21 for amplifications). Significantly higher rates of *ERBB2* alterations were found in female patients (female vs. male, mutations, 2.8% vs. 1.5%, *p* < 0.0001; amplifications, 3.7% vs. 2.1%, *p* < 0.0001). Given the higher incidence of *ERBB2* mutations in breast cancer and gynecologic cancer, the analysis was re-conducted with these cancers being excluded, which showed a consistent higher rate in female patients compared to male patients (3.1% vs. 1.5%, *p* < 0.0001 for SNVs/indels and 2.7% vs. 2.1%, *p* = 0.037 for amplifications).

**FIGURE 4:**
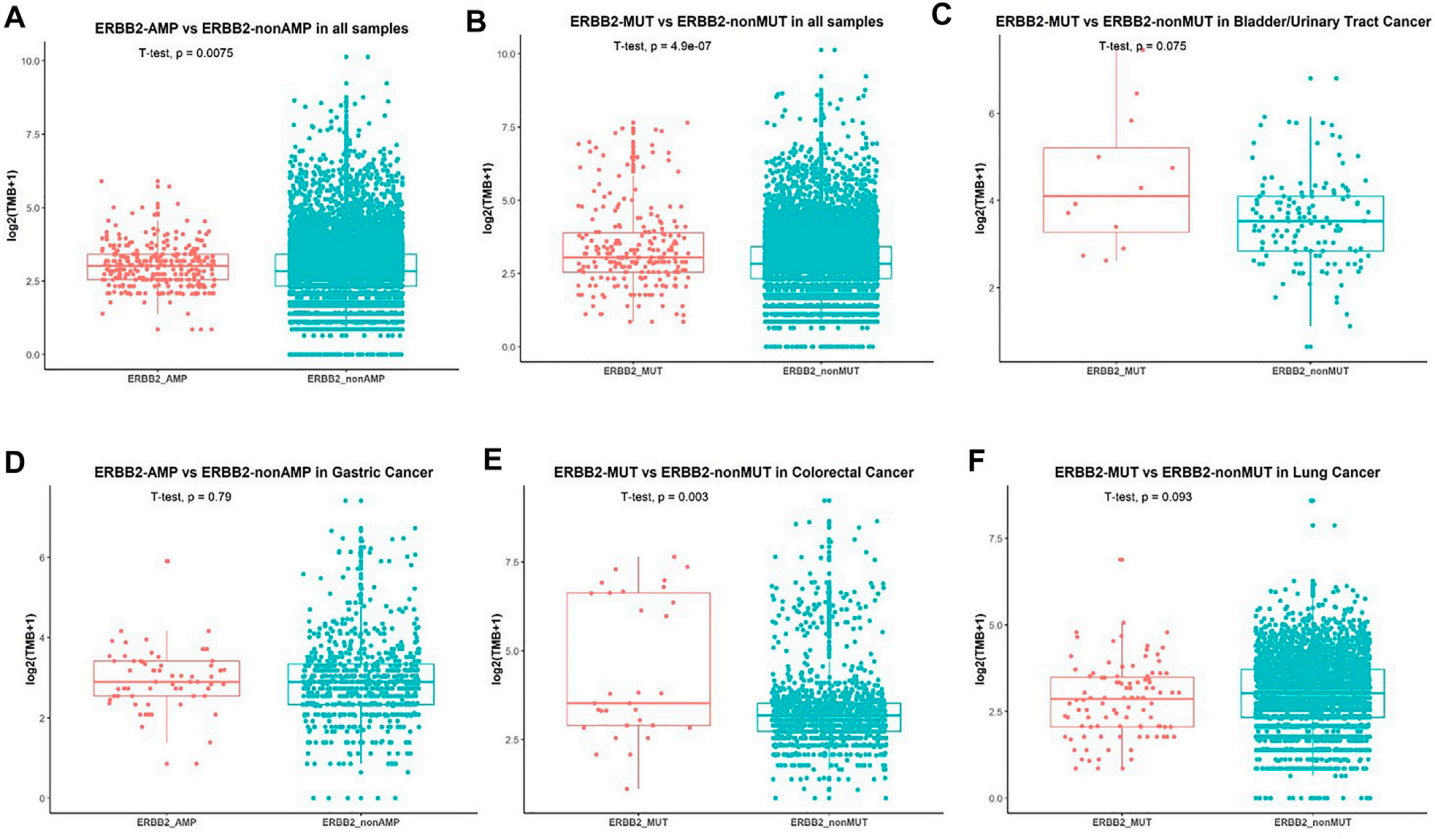
Association between *ERBB2* alterations and TMB. **(A)** Comparison of TMB in patients with *ERBB2*-amplification and non-*ERBB2*-amplification. **(B)** Comparison of TMB in all patients with *ERBB2*-mutation and non-*ERBB2*-mutation and in bladder/urinary tract cancer **(C)**, stomach cancer **(D)**, colorectal cancer **(E)**, lung cancer **(F)**.

We further analyzed the association between *ERBB2* variant and TMB. Patients without *ERBB2* alterations had significantly lower TMB compared with *ERBB2* mutation (*p* = 1.6e^−0.6^) (Figure 4A) and *ERBB2* amplification (*p* = 0.0024) (Figure 4B) respectively in overall population. In a more detailed analysis, we found patients with *ERBB2* mutation had significantly higher TMB compared with non-*ERBB2* mutation in bladder/urinary tract cancer (*p* = 0.0045, Figure 4C), stomach cancer (*p* = 0.0045, Figure 4D), and colorectal cancer (*p* = 0.0021, Figure 4E) respectively. In contrast, patients with *ERBB2* mutation had lower TMB compared with non-*ERBB2* mutation in lung cancer (*p* = 0.048, Figure 4F). However, when we analyzed the association between *ERBB2* amplification and TMB in several cancers, no significant difference was found (Supplementary Figure S1). We speculated that the small sample size in every cancer analyzed may be one reason. On the other hand, *ERBB2* amplification detected by NGS was not totally equal to the status of HER2-positive detected by IHC in breast and stomach cancer. We also explored the relationship between MSI-H and *ERBB2* mutation. Patients accompanying with MSI-H were mostly in the *ERBB2* mutation group (Supplementary Figure S2).

## Discussion

In the present cross-sectional study, we explored the mutational landscape of *ERBB2* in 14,956 pan-cancer patients. Our study showed a varying pattern of *ERBB2* mutations across different tumor types. Overall, the distribution and incidence of *ERBB2* amplifications and mutations in our Chinese cohort were mostly consistent with that in the Caucasian population derived from databases including the Catalogue of Somatic Mutations (COSMIC) and The Cancer Genome Atlas (TCGA) (21–24).

The identification of *ERBB2* amplifications across tumors will provide evidence for the utilization of targeted agents such as trastuzumab, pertuzumab, T-DM1, lapatinib, neratinib, and the trastuzumab biosimilars. A study about HER2-positive breast cancer revealed the relationship between HER2 activity and the pro-trastuzumab tumor immune microenvironment. Gene expression profiling and immunohistochemistry analysis of 53 HER2-positive breast cancer patients indicated that trastuzumab-sensitive tumors expressed significantly higher levels of chemokines involved in immune cell recruitment, with higher infiltration of T cells and monocytes (25). The results were further supported by the recent results from a phase II trial, in which 37 treatment-naïve patients with HER2-positive metastatic oesophagogastric cancer were treated with a combination of pembrolizumab with trastuzumab and chemotherapy, providing numerically higher efficacy (objective response rate, 91%; median overall survival, 27.3 months) compared to the existing first-line standard (chemotherapy plus trastuzumab, objective response rate, 47%; median overall survival, 13.8 months) (8, 26). In this study, we found that patients with *ERBB2* amplification had higher TMB than non-*ERBB2* amplification (*p* = 0.0024), which provided new evidence for the application of immunotherapy in *ERBB2* amplification cancers.

Besides, *ERBB2* exon 20 insertions were found in 0.6% of the entire population and 1.9% of lung cancer patients. The most frequently appearing subtypes were *Y772_A775dup* (82/93, 88.2%) and p.G778_P780dup (8/93, 8.6%). *ERBB2* exon 20 insertions were reported as analogous to *EGFR* exon 20 insertions and associated with primary resistance to currently approved tyrosine kinase inhibitors because of steric hindrance in the drug-binding pocket and a poor response to immunotherapies in lung adenocarcinoma (27, 28) _ENREF_24. In further analysis, *ERBB2* exon 20-mutated tumors exhibited overexpression of RIPK1 and STK11IP and a decrease of cytotoxic natural killer cells (28). We also found lung cancer patients with *ERBB2*-mutant have lower TMB than non-*ERBB2* mutant patients (*p* = 0.048). Taken together, these data suggest that *ERBB2*-mutant lung cancer patients may not benefit from immunotherapy. Whether immunotherapy can be used in other cancers carrying *ERBB2* mutations/amplifications need further exploration.

In conclusion, we revealed a pan-cancer molecular landscape of *ERBB2* amplification and mutations, and patients with *ERBB2* alterations had higher TMB.

## Data Availability

The original presentations in the study are included in the article/Supplementary Material, further inquiries can be directed to the corresponding author. The raw data supporting the conclusion of this article will be made available by the authors, without undue reservation.
