# Supplementary material for: Molecular Landscape of ERBB2 Alterations in 14,956 Solid Tumors
Source: Pathol Oncol Res. 2022 Jul 13;28:1610360. doi: 10.3389/pore.2022.1610360 (PMC9325965; doi:10.3389/pore.2022.1610360)
Supplement: Supplementary file 1 [file DataSheet1.docx]

**Supplementary appendix**

Hao Wang, Ji Miao, Yazhou Wen, et al. Molecular Landscape of ERBB2 alterations in 14,956 solid tumors

**Supplementary Table S1.** Patients with *HER2* mutation in different tumor sites.

**Supplementary Figure1.** Association between *ERBB2* amplification and TMB in breast cancer (A), stomach cancer (B), colorectal cancer (C), biliary tract cancer (D), lung cancer (E).

**Supplementary Figure 2.** Association between *ERBB2* alterations and percentage of MSI-H.

**Supplementary Table S1.** Patients with *HER2* mutation in different tumor sites.

| ***HER2* subtypes** | **Counts** | **Percent** | ***HER2* subtypes** | **Counts** | **Percent** |
| --- | --- | --- | --- | --- | --- |
| **Biliary Tract Cancer** | |  | **Lung cancer** | |  |
| *p.S310F* | 15 | 0.4688 | *p.Y772_A775dup* | 78 | 0.624 |
| *p.S310Y* | 6 | 0.1875 | *p.G776delinsVC* | 10 | 0.08 |
| *gain* | 5 | 0.1562 | *p.S310F* | 7 | 0.056 |
| *p.D769Y* | 3 | 0.0938 | *p.V659E* | 7 | 0.056 |
| *p.R678Q* | 3 | 0.0938 | *p.G778_P780dup* | 5 | 0.04 |
| *p.V842I* | 2 | 0.0625 | *gain* | 4 | 0.032 |
| *p.D277Y* | 1 | 0.0312 | *p.L755P* | 3 | 0.024 |
| *p.D769H* | 1 | 0.0312 | *p.D769Y* | 2 | 0.016 |
| *p.I767M* | 1 | 0.0312 | *p.A1212V* | 1 | 0.008 |
| *p.K684E* | 1 | 0.0312 | *p.A775_G776insSVMA* | 1 | 0.008 |
| *p.S1051C* | 1 | 0.0312 | *p.A775_G776insVVMA* | 1 | 0.008 |
| *p.S202F* | 1 | 0.0312 | *p.G660delinsER* | 1 | 0.008 |
| *p.S653C* | 1 | 0.0312 | *p.G776_V777delinsVVG* | 1 | 0.008 |
| *p.T862A* | 1 | 0.0312 | *p.G776delinsLC* | 1 | 0.008 |
| *p.Y772_A775dup* | 1 | 0.0312 | *p.G776delinsVCC* | 1 | 0.008 |
| **Breast Cancer** |  |  | *p.G776delinsVV* | 1 | 0.008 |
| *p.V777L* | 3 | 0.2727 | *p.G776S* | 1 | 0.008 |
| *gain* | 2 | 0.1818 | *p.G778_S779insCPG* | 1 | 0.008 |
| *p.D769Y* | 1 | 0.0909 | *p.I767M* | 1 | 0.008 |
| *p.G778_P780dup* | 1 | 0.0909 | *p.L181V* | 1 | 0.008 |
| *p.I714T* | 1 | 0.0909 | *p.L755A* | 1 | 0.008 |
| *p.I767M* | 1 | 0.0909 | *p.L869R* | 1 | 0.008 |
| *p.L755S* | 1 | 0.0909 | *p.R103Q* | 1 | 0.008 |
| *p.S310F* | 1 | 0.0909 | *p.R677Q* | 1 | 0.008 |
| *p.S310Y* | 1 | 0.0909 | *p.R896H* | 1 | 0.008 |
| *p.T862S* | 1 | 0.0909 | *p.T862A* | 1 | 0.008 |
| *p.V842I* | 1 | 0.0909 | *p.V777L* | 1 | 0.008 |
| **Stomach Cancer** |  |  | **Colorectal Cancer** | | |
| *p.R678Q* | 20 | 0.4878 | *p.R678Q* | 13 | 0.3514 |
| *p.V842I* | 8 | 0.1951 | *p.V842I* | 9 | 0.2432 |
| *gain* | 6 | 0.1463 | *gain* | 4 | 0.1081 |
| *p.S310F* | 5 | 0.122 | *p.L755S* | 3 | 0.0811 |
| *p.V777L* | 2 | 0.0488 | *p.S310F* | 3 | 0.0811 |
| *p.G776V* | 1 | 0.0244 | *p.V777L* | 3 | 0.0811 |
| *p.H878Y* | 1 | 0.0244 | *p.G776V* | 2 | 0.0541 |
| *p.L317P* | 1 | 0.0244 | *p.S310Y* | 2 | 0.0541 |
| *p.L726V* | 1 | 0.0244 | *p.A1190V* | 1 | 0.027 |
| *p.L755S* | 1 | 0.0244 | *p.A365V* | 1 | 0.027 |
| *p.R896G* | 1 | 0.0244 | *p.E620D* | 1 | 0.027 |
| *p.S310Y* | 1 | 0.0244 | *p.G778_P780dup* | 1 | 0.027 |
| *p.T862A* | 1 | 0.0244 | *p.I767L* | 1 | 0.027 |
| p.V308L | 1 | 0.0244 | p.I767M | 1 | 0.027 |
| **Intestine Cancer** | |  | p.L1131Pfs*8 | 1 | 0.027 |
| p.S310F | 4 | 0.3636 | p.L841V | 1 | 0.027 |
| gain | 2 | 0.1818 | p.Q1225P | 1 | 0.027 |
| p.I767M | 2 | 0.1818 | p.R456H | 1 | 0.027 |
| p.S310Y | 2 | 0.1818 | p.R897Q | 1 | 0.027 |
| p.T733I | 2 | 0.1818 | p.V839M | 1 | 0.027 |
| p.D769H | 1 | 0.0909 |  |  |  |
| p.D769Y | 1 | 0.0909 |  |  |  |
| p.G58R | 1 | 0.0909 |  |  |  |
| p.R678Q | 1 | 0.0909 |  |  |  |
| p.V842I | 1 | 0.0909 |  |  |  |


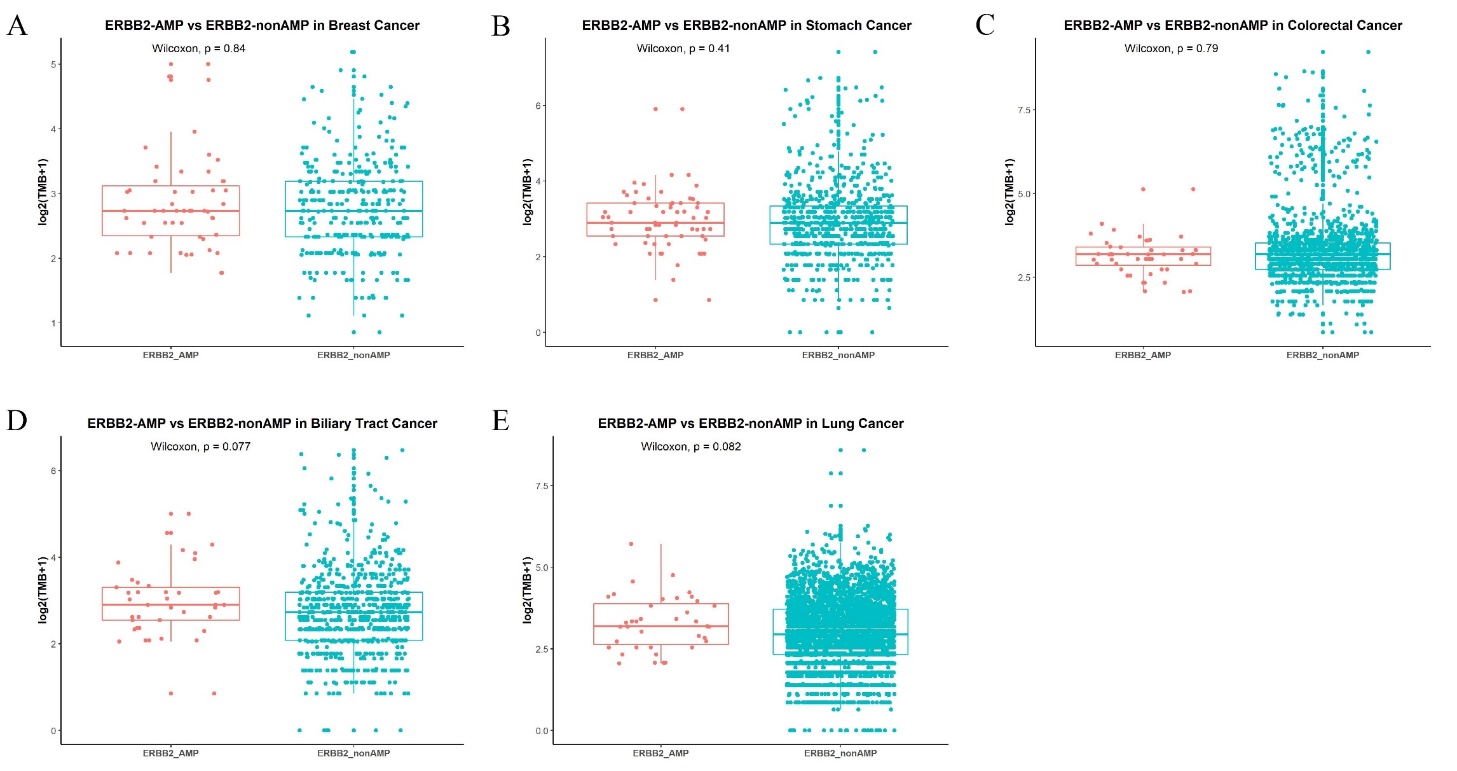


**Supplementary Figure 1**. Association between ERBB2 amplification and TMB in breast cancer (A), stomach cancer (B), colorectal cancer (C), biliary tract cancer (D), lung cancer (E).


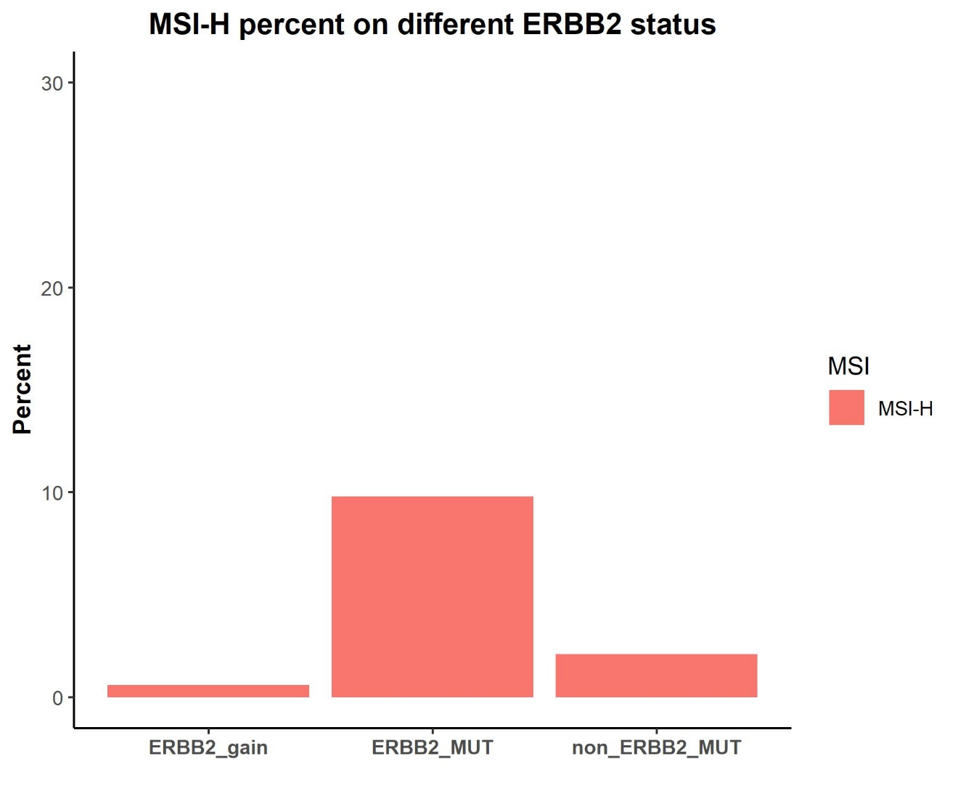


**Supplementary Figure 2**. Association between ERBB2 alterations and percentage of MSI-H.
